# A guide to: generation and design of nanobodies

**DOI:** 10.1111/febs.15515

**Published:** 2020-08-28

**Authors:** Serge Muyldermans

**Affiliations:** ^1^ Cellular and Molecular Immunology Vrije Universiteit Brussel Belgium; ^2^ Liaoning Key Laboratory of Molecular Recognition and Imaging School of Bioengineering Dalian University of Technology China

**Keywords:** immune libraries, naïve libraries, nanobodies, single‐domain antibodies, synthetic libraries

## Abstract

A nanobody (Nb) is a registered trademark of Ablynx, referring to the single antigen‐binding domain of heavy chain‐only antibodies (HCAbs) that are circulating in Camelidae. Nbs are produced recombinantly in micro‐organisms and employed as research tools or for diagnostic and therapeutic applications. They were – and still are – also named single‐domain antibodies (sdAbs) or variable domain of the heavy chain of HCAbs (VHH). A variety of methods are currently in use for the fast and efficient generation of target‐specific Nbs. Such Nbs are produced at low cost and associate with high affinity to their cognate antigen. They are robust, strictly monomeric and easy to tailor into more complex entities to meet the requirements of their application. Here, we review the various sources and different strategies that have been developed to identify rapidly, target‐specific Nbs. We further discuss a variety of engineering technologies that have been explored to broaden the application range of Nbs and summarise those applications where designed Nbs might offer a marked advantage over other affinity reagents.

## Graphical abstract

Immune or naïve nanobody (Nb) repertoires are cloned from B lymphocytes of camelids. Alternatively, synthetic Nb libraries are generated *in vitro* by randomising codons forming antigen‐binding loops of a Nb scaffold. These libraries serve to retrieve target‐specific Nbs by phage display pannings, yeast surface display or other selection methods. After identification of the lead Nb and possible engineering efforts, multiple applications have been explored in research, diagnosis or therapy.

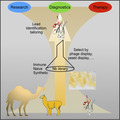

**Abbreviations**
Ag: antigen
BBMV: broad bean mottle virus
CAR‐T: chimeric antigen receptor in T cells
CDR: complementarity determining region
cpDHFR: circularly permutated dihydrofolate reductase
FGE: formyl‐glycine generating enzyme
HCAb: heavy chain‐only antibody
MW: molecular weight
NAR: new antigen receptor
Nb: nanobody
scFv: single‐chain variable fragment
sdAb: single‐domain antibody
SPR: surface plasmon resonance
TEV: tobacco etch virus
TRNT: targeted radionuclide therapy
VHH: variable domain of heavy chain of heavy chain‐only antibody
V‐NAR: variable fragment of new antigen receptor immunoglobulin

## Introduction

**Fig. 1:**
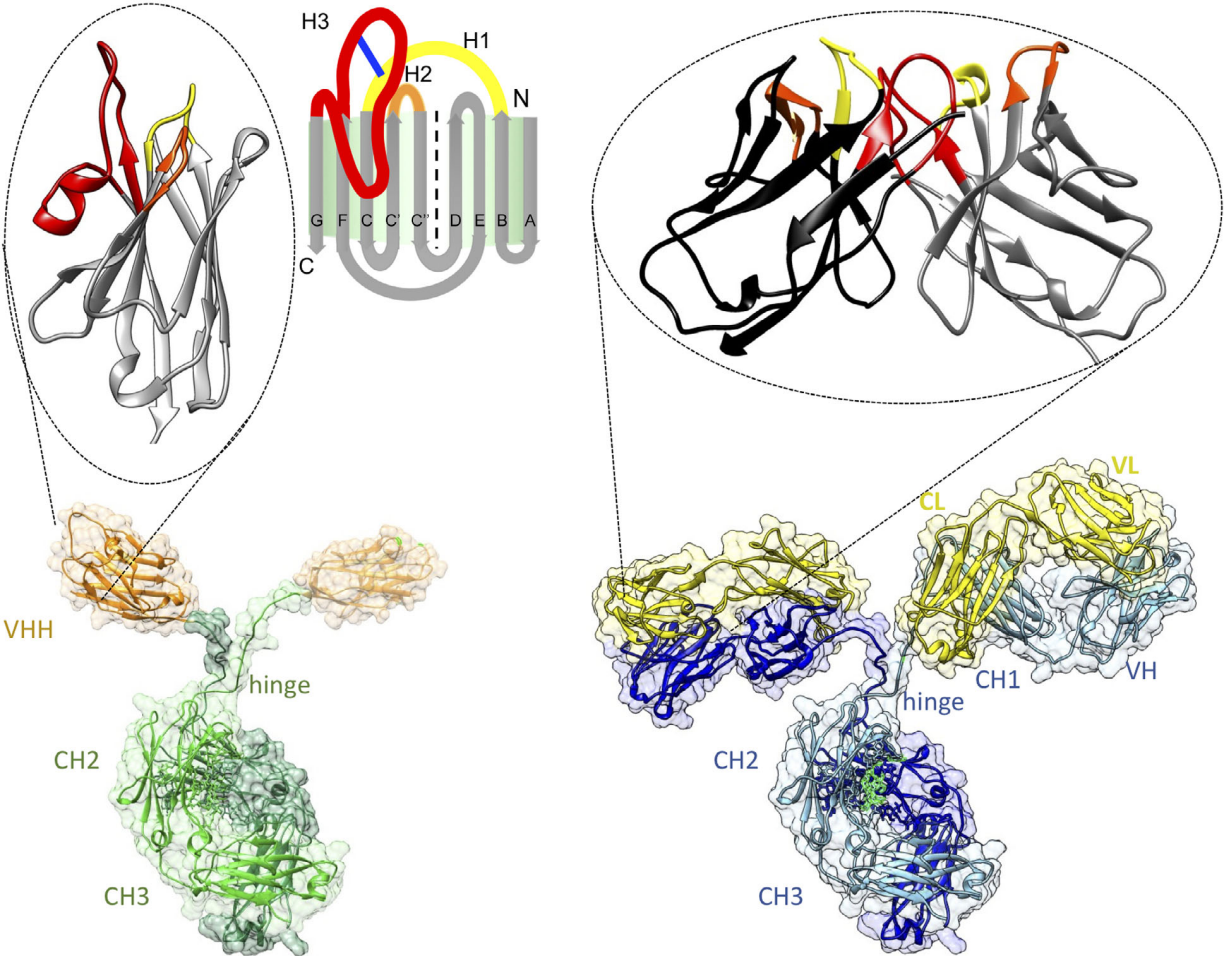
Architecture of homodimeric heavy‐chain antibodies (bottom left) with the Ag‐binding single‐domain VHH, enlarged on top; and classical heterotetrameric antibodies (bottom right) with the Ag‐binding variable fragments (comprising VH and VL domains) enlarged on top. The VHH is in orange; the two heavy chains within the HCAb comprising the hinge, CH2 and CH3 domains are in sea green and olive green. For the classical antibody, the light chains composed of a VL and CL domain are in yellow, the heavy chains with VH, CH1 and Fc (i.e. hinge, CH2 and CH3) domains are in blue and cyan). The Ag‐binding domains (VHH or Fv = VH + VL) have their framework region in grey (VHH and VH) and black (VL) with the Ag‐binding loops in yellow, orange and red for CDR1, CDR2 and CDR3, respectively. A schematic view of the VHH fold with two β sheets is shown at the top, middle. The scaffold β‐strands (grey) named A, B, C, C′, ″, D, E, F and G are indicated as well as the N and C‐terminal ends. Ag‐binding loops named H1, H2 and H3 are shown in yellow, orange and red, respectively (the blue line is the interloop disulphide bond frequently observed in camel‐derived Nbs). UCSF Chimera [149] was used to generate the molecular graphics of pdb 1IGT (classical IgG) and 1JTT (Nb part).

Antibodies are a preferred tool in many applications including research, diagnosis and therapy as they (a) can be elicited against all possible targets; (b) associate with high affinity to their cognate antigen (Ag); (c) are highly specific for their Ag; and (d) can be obtained in a monoclonal form. Unfortunately, their large molecular weight (MW: 150 000) and heterotetrameric composition with two different polypeptides (a heavy and a light chain) and a total of close to 15 disulphide bonds that have to be formed often under unfavourable redox conditions prevent a facile production in bacteria or in the cytoplasm of eukaryotic cells. In this regard, the single‐chain variable fragment (scFv), comprising the variable domain of the heavy and of the light polypeptide chain joined with a synthetic linker into a single polypeptide chain [1] and forming the intact Ag‐binding site of the antibody [2], offers several advantages (Fig. 1). For example, the genetic engineering toolbox is more adapted to such smaller constructs (MW scFv ~ 30 000) and a faster and more economic production in bacteria becomes feasible. Moreover, with the development of the phage display technology [3], an extremely powerful selection technology became available to retrieve, in a few weeks' time, multiple potent affinity reagents from a large and diverse collection of scFvs [4]. Although this boosted the use of scFvs [5], their fragile behaviour leading to denaturation and/or aggregation or a spontaneous dimerisation into diabodies remained an apparent weakness that complicated further applications. Therefore, scientists started early on to substitute scFvs by manmade single‐domain protein scaffolds that could be randomised at one side of the domain to generate diversity and to construct synthetic libraries from which to retrieve potent affinity reagents, mostly by phage display or ribosome display. Multiple protein scaffolds were developed, including affibodies based on a monomeric subunit domain of protein‐A, anticalins using lipocalins, monobodies or Adnectins based on a fibronectin domain, a carefully selected human VH domain engineered for increased stability and solubility, knottins based on small cysteine‐rich proteins with a knotted topology and designed ankyrin repeat proteins [6, 7, 8]. All these scaffolds share a monomeric, soluble, stable fold with a small size and MW ranging from 7000 to 18 000, a good expression level in micro‐organisms and an expected low immunogenicity when administered in humans [9]. The properties of variable domain of the heavy chain of heavy chain‐only antibodies (VHH) or nanobodies (Nbs) also fit into this list of desired properties, and they have the additional advantage to originate from *bona fide* homodimeric heavy chain‐only antibodies (HCAbs) devoid of light chains, circulating in Camelidae (Fig. 1) [10]. Hence, the natural function of HCAbs in camelids is to target Ags, their genes have evolved over millions of years for this purpose, and they can be affinity‐matured, *in vivo*, in a short time by immunising the animal [11, 12]. Besides camelids, also the immune system of some sharks seems to possess HCAbs, referred to as immunoglobulin new Ag receptor (NAR), that recognise the Ag with one single variable domain, known as variable domain of NAR (V‐NAR) [13]. The V‐NARs and VHHs share many properties, such as a preferential binding to cavities or grooves on the surface of the Ag and resistance to extreme conditions (pH, pressure, chaotropic agents, temperature or proteases) often assisted by an extra interloop disulphide bond [14]. However, their structural architecture deviates slightly. The VHH is very similar to a (human) VH comprising 2 β‐sheets, one with 4 and one with 5 β‐strands (Fig. 1), with the three Ag‐binding loops between β‐strand B–C, C′–C″ and F–G [15]. In contrast, the V‐NARs resemble rather a constant immunoglobulin domain with poorly pronounced C′–C″ strands and loop [14]. In addition, while amino acid sequences of VHH and human VH are very similar [16, 17], the sequence divergence between V‐NAR and mammalian variable immunoglobulin domains such as VHH or human VH is obviously much larger [18, 19, 20].

In the early days, when the recombinant antibody engineering technology – mainly focusing on scFvs – emerged, Nbs did not attract much attention. However, over the years, a steadily growing number of publications illustrated the benefits of Nbs in particular niches of research [21]. To identify practical Nbs, it is important to start from a high‐quality gene bank of Nbs, dedicated to the final application and with maximal diversity. We then need a suitable selection procedure to retrieve the best possible Nbs from this diverse library and possibly, minor engineering to tailor the Nb for its final application. Nowadays, the technology to identify Nbs tailored into practical constructs is offered by several small biotech companies and by service facilities in many academic institutes. Even big pharma industry has discovered the benefits of Nbs [22]. This exponentially growing number of Nb users makes that the number of dedicated applications is exploding over the last decade. Below we summarise the various types of Nb libraries (Table 1) that are in use to retrieve target‐specific Nbs, and we highlight the adequate technologies to retrieve the best possible Nbs from these libraries that can be employed – possibly after minor tailoring – in a fast‐growing number of applications. We highlight those applications where the beneficial properties of Nbs might make a significant difference (Table 1).

**Table 1:** Overview of the various types of Nb libraries with their specifications, the Nb selection, the primary and secondary features of the identified Nb and a list of possible applications where Nbs might make a difference.

Nb library	
Immune	Start with blood of an immunised camelid
	Multiple libraries will be needed for various projects
	(Relatively) Small library size (10^6^–10^8^)
	Affinity‐matured Nbs will be obtained
	(Relative) high titre of target‐specific binders
Naïve	Immunisation is avoided (no adverse effect from toxic targets)
	Works for nonimmunogenic targets as well
	Very large library size (10^9^–10^11^) is needed
	One library serves for multiple projects
	Nbs might require stability/affinity improvement
Synthetic	Immunisation is omitted (no adverse effect from toxic targets)
	Works for nonimmunogenic targets as well
	Extremely large library size (10^9^–10^15^) is required
	One library serves for multiple projects
	Nbs might require stability/affinity improvement
Nb selection	
Phage display	Robust, fast, many selection tricks, versatile
Yeast display	Highly selective, FACS gating for special function
Bacterial display	Sporadically used
Two hybrid	Bacterial or yeast, sporadically used
Ribosome display	*In vitro* technique, sporadically used for Nbs
CIS display	*In vitro* technique, sporadically used
Deep sequencing	Sporadically used
Phenotypic selection	Highly promising for selecting function modulating Nbs
NestLink based selection	Amenable to sophisticated screenings, for example panning in organisms
Nb properties	
Primary features	Secondary features
Small size/low MW	Fast blood clearance
Economic production	Deep penetration in tissues
High specificity	Enzyme modulating
High affinity	Low immunogenicity (well tolerated and nontoxic)
Robust	Long shelf life
Monomeric	High solubility
Easy engineering	Directional immobilisation
Genetically encodable	Can be splitted and integrated in other protein scaffolds
Nb applications	
Research	Target crystallisation chaperon
	Superresolution microscopy tool
	Intracellular expression/intrabodies
	Intracellular target tracing; interference with endogenous target
	Interference with *in vivo* function of endogenous Ag
	Developmental biology
	Manifold constructs (bivalent/biparatopic/multivalent/bispecific)
*In vitro* diagnostic	Lateral flow assays
	Electrochemical Ag detection
*In vivo* diagnostic	Noninvasive *in vivo* imaging
Therapy	Autoimmune disease and inflammation
	Cancer
	Infectious diseases
	Envenoming
	Immuno‐pheresis
(Agro‐)Biotech	Immuno‐adsorbents
	Protection of plants against pathogens
	Animal feeding

## Generation of nanobody libraries

### Immune, synthetic and naïve libraries

Three types of Nb banks can be employed to retrieve Ag‐specific Nbs, the so‐called immune, naïve and synthetic library (Table 1). For an immune library, we first have to immunise a young adult, healthy Bactrian camel, dromedary, llama or alpaca. In principle, a vicuña or guanaco could also be used for immunisation as they also contain HCAbs, but these are wild animals that should not be used for laboratory tests. Typically, in a time span of 2 months, animals are injected four to eight times with the target Ags, mixed with a standard adjuvant to vaccinate cattle or sheep. We recommend using about 50–200 μg of immunogen per injection; the exact amount obviously depends on the MW of the Ag and even more on its immunogenicity and/or toxicity. Soluble, properly folded recombinant proteins are preferred for the immunisation, although DNA vaccination also has been very successful [23, 24]. We usually mix up to 10 proteins to immunise an animal, but even more complex mixtures seem to work (i.e. viruses, bacteria, parasites, intact mouse splenocytes or protein extracts of cancer cells [25, 26, 27, 28, 29]. Small molecules (haptens) or oligopeptides are poorly antigenic for HCAbs. Nevertheless, some notable exception has been reported for structured oliopeptides [30] and small organic molecules [31, 32], although unusual Nb or hapten modifications might have occurred [31, 33]. In cases where Nbs are desired that cross‐react with the human and mouse homologues, it is advisable to boost the immune response of the camelid alternatively with the human immunogen and its mouse equivalent. To increase the likelihood to obtain Nbs against predesignated epitopes, it is recommended to immunise more than one animal. Since they are outbred animals, every animal will raise a unique immune response and a larger panel of Nbs will be obtained, from which to choose the best performing Nb. Of note, the ratio of HCAbs over classical antibodies is larger in camels or dromedaries compared to llama and alpaca [34]. This suggests that a larger variety of Nbs might be retrieved from an immunised camel than from immunised Laminae.

Apart from immunising a camelid, several groups have invested in generating a transgenic mouse producing HCAbs. Obviously, these transgenic mice are a good substitute for camelids in cases where the Ag is difficult to obtain, and indeed, good single‐domain antibodies (sdAbs) have been retrieved following immunising such mice [35, 36].

**Fig. 2:**
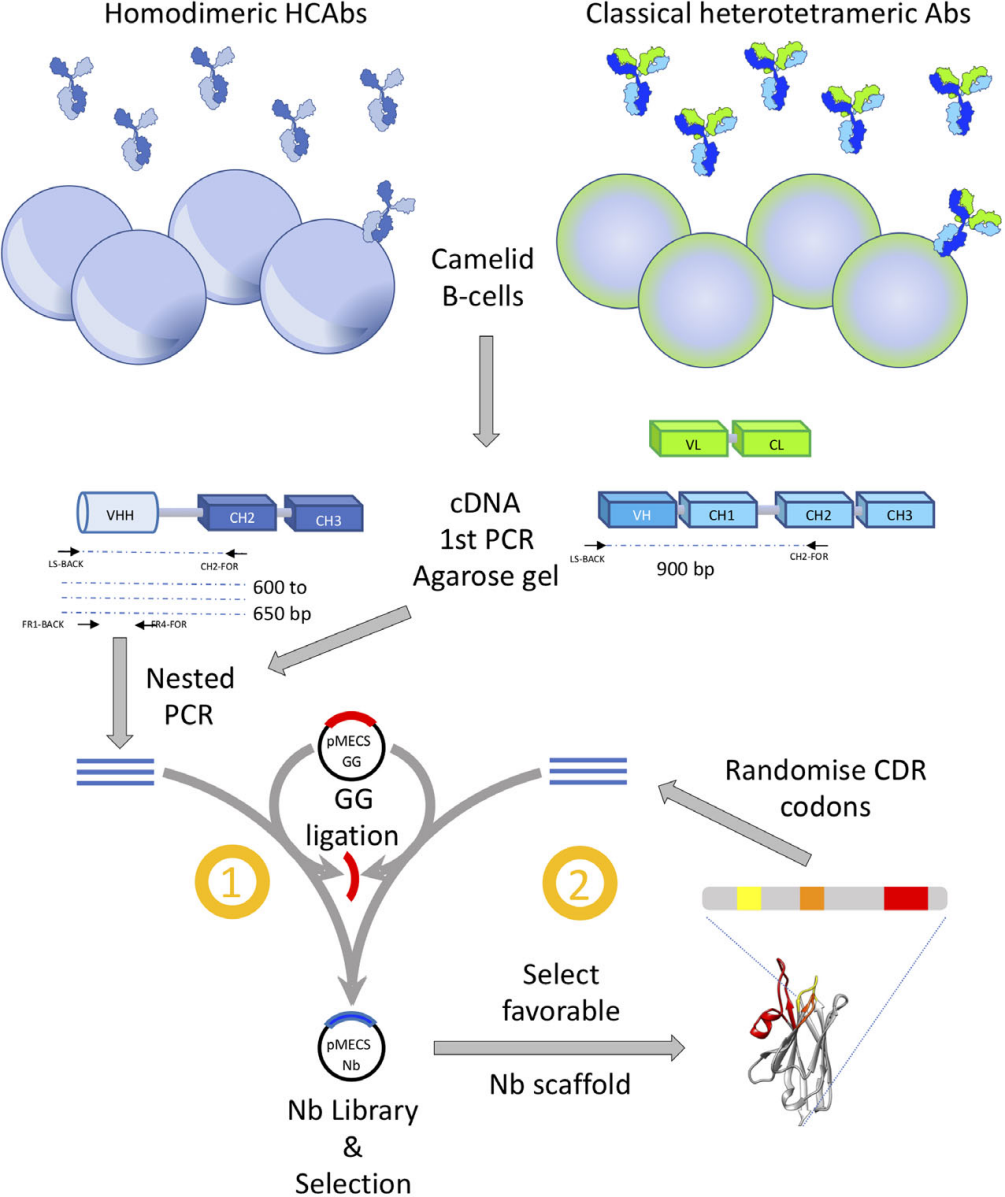
Schematic overview to generate immune, naïve, and synthetic Nb libraries. Blood in camelids contains two kinds of B cells that secrete either homodimeric HCAbs or classical heterotetrameric antibodies. The mRNA of these B cells is converted into cDNA and a first PCR with primers annealing to the leader signal and conserved CH2 region (LS‐BACK and CH2‐FOR, respectively) on this cDNA will amplify a fragment of 600–650 bp with VHH‐hinge‐CH2 coding sequences of HCAbs and a fragment of 900 bp with VH‐CH1‐hinge‐CH2 sequences from the heavy chain of classical antibodies. The former amplicon is purified after agarose gel electrophoresis and amplified in a second, nested PCR with primers annealing at framework region 1 and 4 (FR1‐BACK and FR4‐FOR, respectively). This PCR amplification product is ligated into the, for example pMECS‐GG phagemid vector (procedure “1”) to generate pMECS‐Nb phagemids that are transformed in *Escherichia coli*. An immune Nb library (typically 10^6^–10^8^ clones) is obtained when starting from a 50 to 100 mL blood sample of an immunised camelid, whereas a naïve Nb library (typically 10^9^–10^11^ clones) is generated when starting from much larger amounts of blood (~ 10 L) taken from several healthy camels, dromedaries, llama's or alpaca's that were not intentionally immunised. To construct a synthetic Nb library (procedure “2”), a single or a few Nbs are expressed form the previous libraries and selected for favourable (biochemical) properties (stability, expression level, solubility, unique paratope architecture). The codons for amino acids located in the Ag‐binding loops (yellow, orange and red for CDR1, 2 and 3) of these selected Nb scaffolds are then randomised and the sequences amplified by PCR. These PCR products are ligated, for example in phage display vector pMECS‐GG to obtain a synthetic pMECS‐Nb library after transforming *E. coli* cells.

After the immunisation, a small aliquot of 50–100 mL anticoagulated blood is taken, usually from the jugular vein – although a lymph node biopsy is also a good starting material [37] ‐ to prepare lymphocytes and to extract mRNA. The mRNA is converted into cDNA and used to amplify the VHH gene regions. This is most efficiently achieved in a two‐step nested PCR (Fig. 2) [38]. In the first PCR, we amplify the heavy chain of all IgGs from the leader sequence to a conserved region within the CH2 exon (Fig. 2). This PCR amplifies the ‘VH‐CH1‐hinge’ encoding cDNA from classical antibodies and the ‘VHH‐hinge’ encoding cDNA from HCAbs. The latter amplicons have a smaller size since the CH1 exon is absent. These smaller amplicons are easily purified after agarose gel electrophoresis and used as template in a second PCR to amplify the VHH with primers containing suitable restriction enzyme sites. There have been reports where the elimination of the VH sequences has been omitted and such libraries yielded useful binders as well [39]. However, this strategy is less ideal as the VH sdAbs in absence of VL domains might be sticky, which might complicate the subsequent enrichments of Ag‐specific Nbs. Interestingly, all *VHH* genes cluster with the human family‐3 *VH* genes in a phylogenetic analysis [17, 40, 41, 42]. Therefore, it is possible to amplify all *VHHs* with one single set of PCR primers. In contrast, since human *VH* genes belong to five or six families and each family requires dedicated primers for its PCR amplification, multiple primer mixtures need to be tested. Remarkably, the camelid genome also contains a *VH* gene corresponding to human *VH* of family‐2 that can be recombined with *D* and *J* genes for usage in both, classical antibodies and HCAbs [42, 43]. However, since the HCAbs equipped with a *VH* of family‐2 form only a minority in the IgG repertoire, it is acceptable to neglect these Nbs and to exclude them from the immune Nb library. The ends of the PCR amplicons are digested with restriction enzyme, ligated in the appropriate vector and transformed in *Escherichia coli*. A good immune Nb library should contain > 10^7^ individual transformants, and at least 70% of the clones should contain a plasmid with an insert with the size of a VHH. Following the recent successes of scFv and nonimmune Nb (Sybodies) libraries [44, 45], we introduced successfully the Golden Gate technology to increase the percentage of clones with plasmids containing a VHH insert [46]. Since the type IIs restriction enzyme digestion and ligation occur simultaneously to replace a toxic gene in the phage display vector, we reach libraries with close to 100% of the clones containing a VHH gene. A few randomly chosen clones of the constructed library are sequenced to ensure that the inserts are encoding an in‐frame, putative functional VHH. However, the sequencing results of this limited number of clones cannot serve to claim features of the VHH diversity within the library. The VHH diversity can only be properly investigated after a deep sequencing effort.

There is pressure to circumvent the immunisation of animals because alternatives are available [47]. Moreover, some molecules such as RNA or DNA are not immunogenic, or at least fail to elicit an immune response in the HCAb classes, while other compounds might be too toxic, too contagious or too harmful for animal or environment. In those cases, the construction of a naïve Nb repertoire or a synthetic Nb repertoire might be envisioned [48, 49, 50]. Once such single‐pot libraries are available, the time to identify first‐generation Ag binders against virtually any target can be shortened to < 2 weeks. To retrieve high‐affinity binders from such naïve or synthetic Nb libraries, it is well accepted that library sizes of 10^9^–10^10^ individual clones should be aimed at, of which > 80% should encode a Nb. To construct such a large and diverse, naïve Nb library, one should start from a large pool of blood taken from several animals (blood from at least 10 animals should be taken, to avoid a bias in HCAbs due to an allergy or from a previous infection). With an estimated 10^6^ lymphocytes per mL of blood [34] and considering that only a fraction of these are B cells of which ~ 50% might be expressing HCAbs, it is clear that > 10 L of blood will be required to construct a diverse Nb library of 10^10^ different VHH clones. Therefore, the generation of a naïve Nb library seems to be very tedious. In contrast, a naïve scFv library is not dependent on these large volumes of blood, because the VH and VL are first amplified independently from each other and are subsequently combined randomly in a scFv construct. Furthermore, for human naïve scFv libraries, one usually prefers to amplify the VHs from IgM molecules with specific primers, to avoid an overrepresentation of VHs from proliferated and affinity‐matured IgGs [5]. Since there are no – or extremely few – IgM HCAbs, it is not feasible to construct a ‘naïve’ Nb library. Thus, we are bound to go for the target‐dedicated, affinity‐matured and biased IgG HCAbs. Nevertheless, several reports claim to possess over adequate naïve Nb libraries from which Nbs of high affinity could be retrieved [48, 51, 52, 53].

In view of the limitations of naïve Nb libraries, the construction of highly diverse synthetic Nb libraries is probably more worthwhile. Indeed, several synthetic Nb libraries have been reported from which potent Nbs have been retrieved that were used in a variety of applications [44, 54, 55, 56]. The strategy to construct good synthetic libraries is common (Fig. 2): a stable and well‐expressed sdAb scaffold is selected, preferably one for which the crystal structure is available. The Kruse library was synthesised using the consensus sequence of llama germline IGHVS1‐S5 genes [55]. The selection of a consensus Nb sequence is also a method to obtain a more stable scaffold structure. The amino acid diversity is concentrated in the three complementarity determining regions (CDRs; or in the near vicinity of the CDRs). The randomisations within CDR1 and CDR2, will not use all amino acids, cysteine, proline, methionine, are avoided for their chemical reactivity or constraints in folding; isoleucine and leucine are underrepresented to decrease the surface hydrophobicity. Furthermore, alanine, serine, threonine, tyrosine and asparagine are preferred as these amino acids occur frequently in the paratope where they are involved in Ag interaction. Alternatively, at each position within the CDR, codons are included that are most frequently occurring at that position within sdAbs. This is to ensure that only amino acids are inserted that are compatible with proper folding. Finally, since the CDR3 length is highly variable in Nbs, the synthetic library will be constructed using different CDR3 lengths [56, 57]. A different CDR3 length might also lead to a different paratope architecture. To synthesise the mutagenic primers, it is recommended to use the desired mixtures of the trinucleotide building blocks to avoid stop codons, unwanted amino acids and reading frame shifts. Moreover, it allows to obtain the exact amino acid diversity at the sought frequency. Since many codon positions are randomised and substituted by several codons, it follows that the theoretical diversity is always several orders of magnitude larger than the number of clones that will be generated. This has the advantage that each clone within the library will be unique. The quality control of the library will involve several parameters such as the number of transformants, the percentage of intact inserts, the sequence analysis to confirm the chosen randomisations, the production capacity of a number of clones and above all the success rate of retrieving practical binders. The calculation of the success rate percentage is only relevant if the number of widely different targets tested approaches 100 or so. Moreover, not all retrieved Nbs can be considered as ‘binders’ since many will fail to meet the stringent requirements of the particular application that the investigator was envisaging. Finally, since the theoretical size of the library is many times larger than the number of clones screened, each selection will yield different binders of variable binding characteristics. For these reasons, it is really hard to define the ‘quality of a library’. On an interesting note, similar thoughts on the quality of libraries are also valid for immune libraries.

### Retrieval of target‐specific nanobodies

Several selection technologies have been developed to retrieve Ag‐specific Nbs from a library [5]. Phage display, originally developed by George Smith [3] and adapted for scFv by Sir Greg Winter [4], is the oldest and probably still the most robust of these selection technologies. Davies & Riechman applied the phage display technology to retrieve Ag‐specific sdAbs from their ‘camelised’ human VH synthetic library [54]. Around the same time, the technology was also employed to retrieve the first Ag‐binding sdAbs from an immune library [58]. The success rate of identifying Nbs from an immune library is close to 100%, if a properly folded protein was used as immunogen. The combination, immune library and phage display panning are by far the most common method whereby Nbs against > 1000 different Ags have already been isolated and characterised [59]. The phagemid system originally introduced by Hoogenboom *et al*. [60] is most frequently used as cloning vector. After growing a representative aliquot of the library, the cells are infected with M13 helper phages to produce phage particles that contain the Nb coding sequence inside the virion and the corresponding Nb protein fused to Protein‐3 at the tip of phage particle. Incubating these virions in an immunotube (or a well of microtitre plate) with immobilised Ag allows to withhold those virions carrying an Ag‐specific Nb while removing the excess, nonbinding phage particles. A pH shock releases the adsorbed phage particles, which can then be rescued by infecting fresh bacteria via their F pilus. For immune libraries, one to four rounds are sufficient to identify the binders within a time span of 1–2 weeks. Although the passive coating of the Ag on the plastic of wells of microtitre plates or immunotubes works well for most proteins, fragile targets might require a panning in solution for example by biotinylating the Ag and capturing on streptavidin‐coated magnetic beads. This strategy has also the extra benefit that it becomes possible to screen the binders on their equilibrium association constant by reducing the target concentration in the consecutive rounds of panning [5]. Selection of binders with best dissociation rate constant is accomplished by increasing the stringency (or time) of the washing steps during consecutive rounds of panning.

Obviously, the titre of high‐affinity, Ag‐specific (i.e. affinity‐matured) Nbs within a small immune library is higher than that in much larger synthetic or naïve Nb libraries. Therefore, even inexperienced investigators will be successful in retrieving practical Nbs from immune libraries, while more expertise and supervision will be required to obtain useful Nbs from the nonimmune libraries. With large nonimmune libraries, the amount of cells (or fraction of the library size) used to start the culture, the culture volume, the time of infection with helper phages, the handling of fragile virions, the amount and concentration of Ag adsorbed on solid surface or in solution are becoming much more critical parameters. Furthermore, the increased titre of Ag‐specific Nbs within immune libraries contributes to the successful development of phenotypic screening strategies. This has been extremely helpful to identify Nbs inhibiting viral proliferation or to screen for Nbs that interfere with an intracellular reaction pathway [61, 62, 63].

The presence of an amber stop codon between the Nb and the Protein‐3 gene in the phagemid selection system offers the advantage that avidity effects during selections are avoided [60]. In addition, a large amount of ‘bald’ phages are secreted from the bacteria, and since M13 phages are intrinsically sticky, these reduce the efficiency of the enrichment of virions that express target‐specific Nbs. In this regard, the bacterial or yeast surface display systems seem to be superior [55, 64]. It is common knowledge that yeast display selection systems manage to retrieve very good binders even from (relative) small Nb libraries. This might be due to poor expression of Nbs on the virions generating many bald virions when working with phagemids and to the fragility of the extremely long and very thin virions (550 nm by 6 nm for a phagemid vector of 5000 base pairs) that are damaged during handling, thereby loosing infectivity. The selection for yeast display and bacterial display is preferably performed by FACS or MACS [64, 65, 66]. The selection by FACS relies evidently on the availability of an expensive instrument and fluorescently labelled Ags, which involves some extra steps. However, FACS selection has the advantage that we can normalise for Nb expression on the membrane surface [67]. Another advantage of yeast or bacterial surface displays in combination with FACS screening is their amenability to multiplexing whereby the Nb expressing cells can be stained with Ags or confomers, labelled with different fluorophores. Likewise, mixing two proteins (each labelled with a different fluorophore) with the Nb surface‐displaying cells allows to sort those Nbs that recognise each protein independently as well as those Nbs that recognise and stabilise the paired target proteins. Furthermore, once the sorting is finished the cells can just be put in culture medium to proliferate, there is no Ag elution, neutralisation or infection step required as with phage display. Unfortunately, the yeast and heavily engineered bacterial display strains are growing slower compared to customary bacteria producing M13 virions.

Many other selection systems have been proposed and utilised for Nb enrichment and identification. For example, the ribosome display method seems to be suitable to screen libraries with up to 10^14^ variants. Moreover, although the recombinant and optimised PURExpress kits for ribosome display are commercially available, this selection method remains technically demanding [68]. However, a synthetic library enriched by ribosome display is available for academic research from the Seeger laboratory [44, 57]. Besides the *in vitro* ribosome display selection, also the CIS display technology has been employed for selecting Nbs and/or improving existing Nbs [69].

The deep sequencing of immune libraries could in principle be used to identify potential binders [70]. The rationale is that B cells with superior HCAbs will proliferate faster during an immunisation or infection, and the VHH of those B cells will be more abundant in the sequenced repertoire. However, the proliferation of B cells will also depend on the immunogenicity of the Ag, and therefore, the most frequently occurring Nb sequences might not always correspond to the target‐specific Nbs or to the Nbs that bind the Ag with highest affinity. The identification of Nbs directly by mass spectrometry or by bacterial and yeast two hybrid systems has also been proposed [71, 72, 73]. No doubt that these selection methods are valuable under specific conditions, for example the bacterial two hybrid selection is useful to identify Nbs from an immune library, which will be used subsequently as intrabodies. Nevertheless, these selection technologies will never become the gold standard technology for daily Nb generation tasks.

After a few rounds of enrichment, individual clones will be cultured in minimal volumes, their Nb will be expressed and tested, most likely in an ELISA, to identify those clones that produce a Nb recognising the target [74]. Of note, unless the concentration of the Nb is normalised, the intensity of the ELISA signal is a function of both, the expression level of the Nb and the affinity of the Nb–Ag interaction. To identify and eliminate the weakest binders, it is recommended to rank the Nbs' performance by surface plasmon resonance (SPR) or biolayer interferometry. For example, flowing crude Nb extracts over an SPR chip with an immobilised Ag, and estimating the *k*_off_ value during washing with equilibration solution provides valuable information. Since most Nbs bind their Ag with a k_on_ rate of 10^5^–10^6^ m ^−1^^·^s^−1^, a measured *k*_off_ rate of 10^−3^·s^−1^ corresponds to a binding event in the low nm affinity range.

Recently, an innovative selection and identification technology, named NestLink, was developed [75]. In this approach, a Nb library is ligated to genetically encoded barcoding peptides. A representative fraction is deep‐sequenced and also expressed as soluble proteins for mixed with the Ag. The Nb–Ag complex is purified by size exclusion chromatography and the barcode peptide is cleaved from the Nb by proteases for identification by mass spectrometry. Capturing and washing of the Nb‐Ag complex and scoring the barcode frequency of occurrence in washed and retained fractions allowed the ranking of the binders with best *k*_off_ rates. Interestingly, this soluble monomeric barcoded Nb library opens the possibility to perform *in vivo* panning in whole organisms.

However, in the standard phage display procedure, the Nb nucleotide sequence of the ELISA‐positive clones is determined and translated into amino acids. This allows to identify the unique clones and to group the sequences to their CDR3 homology. For Nbs retrieved from immune libraries, those with an identical CDR3 length and a homologous CDR3 sequence are grouped in one family. The Nbs from a family might differ in their CDR1 and/or CDR2 sequences and might also contain several point mutations in their framework regions. The Nbs belonging to one family are derived from the same B cell lineage, that is originating from the same *V‐D‐J* rearrangement and might have been diverged afterwards from each other by somatic hypermutations or gene conversion events during affinity maturation [10]. However, it cannot be excluded that some of the differences were introduced by PCR errors and PCR cross‐overs. Nevertheless, since the Ag recognition is dominated by the CDR3 of the Nb, it has been repeatedly demonstrated that Nbs from the same family will bind to the same epitope on the Ag, although the affinity could be slightly different, due to the mutations among the Nbs of the same family [76, 77].

Finally, all unique Nbs should be expressed, most likely in bacteria, and further characterised in view of the envisaged applications.

## Engineering of the lead Nb to fit the application

### Humanisation of Nbs

For therapeutic application, it became standard practice to humanise the lead Nb to avoid possible immunogenicity issues upon administration in humans. During this humanisation effort, it is best to preserve the four VHH hallmark amino acids (predominantly F/Y42, G/Q49, R50, G52) located in the framework region 2 [78]. Unfortunately, the other amino acids that should be mutagenised to obtain a sequence that is mimicking a human VH sequence are spread throughout the gene. Instead of designing mutagenic primers and a complex assembly strategy, it is much easier to order a synthetic entirely humanised Nb coding sequence taking into account the preferred codon usage of the expression host.

### Genetic fusion with Nbs

Nbs are routinely fused to His6, c‐myc or haemagglutinin tags for purification and detection purposes. The headlock tag (also Spot‐Cap, Chromotek, Planegg‐Martinsried, Germany), C‐tag (ThermoFisher Scientific, Waltham, MA, USA) and ALFA tag (Nano Tag, Göttingen, Germany) are suitable commercial tags against which dedicated Nbs are available for target purification and detection [30, 79, 80]. The ‘Avi’ tag for *in vivo* biotinylating is also frequently used [74, 81].

Very often, the lead Nb needs additional tailoring to fit a particular application. The genetic fusion of the lead Nb coding sequence with another gene is very efficient and provides exactly the designed construct upon expression. The Nb is preferably kept at the 5′ end of the construct so that the N‐terminal end of the Nb will be solvent exposed, which is important since the paratope is also located at this end of the domain. The fused genes are regularly spaced by a linker, where (Gly_4_Ser)_3_ for its enhanced flexibility between the fusion partners or the natural hinge of human IgA for its protease resistance are favoured spacers [1, 81].

The fusion of the coding sequences of two identical Nbs generates a bivalent construct that will show most likely an enhanced functional affinity due to avidity effects [82]. Fusion of two Nbs, binding to nonoverlapping epitopes on the same Ag, will form a biparatopic Nb construct forming a clamp on the Ag [83]. For optimal clamping, it might be necessary to test different linker lengths and to clone the Nbs in different order. The fusion of two Nbs recognising different Ags will bring the two cognate Ags in proximity, which is necessary for bispecific T‐cell engagers (BiTEs) where, for example, a Nb against CD19 of B cells is fused with an anti‐CD3 Nb targeting T cells [84]. Alternatively, the therapeutic Nb might be fused with a second Nb binding to serum albumin. Such bispecific constructs will exhibit an enhanced blood circulation time [85, 86, 87]. Since each Nb behaves independently, also larger multivalent constructs have been assembled, like trimeric or tetrameric Nb constructs [87]. Although these larger constructs will bind with higher efficacy to their target, it comes at an expense of a lower production yield [86, 87]. Therefore, fusion of a Nb with a pentamerising protein will spontaneously generate pentavalent or even decavalent constructs if a Nb was inserted at the N and at the C‐terminal end of the scaffold [88]. On a similar vein, fenobodies have been created whereby a solvent‐exposed helix of ferritin is substituted with a Nb [89]. After expression, the chimeric protein will assemble in a 24‐mer, exposing clustered Nbs resulting in a dramatically increased avidity.

For ELISAs, the Ag‐specific Nb coding sequence has been fused to the cDNA of alkaline phosphatase or horseradish peroxidase [90, 91]. These recombinant fusion proteins can be used directly as detection agent in ELISA but also in electrochemical sensors. The gene construct of a Nb with a fluorescent protein such as monomeric RFP or GFP, expressed intracellularly, was instrumental to generate chromobodies that track the Nbs' Ag in living cells [92, 93, 94]. For therapeutic applications, the Nb coding sequence has been fused to the gene of proteinaceous toxins or has been reconstituted with the hinge and Fc region, mostly of human IgG1 to trigger natural immune effector functions and/or to increase blood circulation time [95, 96].

Furthermore, the intracellularly expressed genetic fusions of Nbs to the F‐box, the von Hippel–Lindau protein, transcription activation domains, DNA binding domains, etc, have generated a variety of exquisite Nb‐based intrabody tools. With these tools at hand, intracellular protein targets have been degraded specifically or genes can be activated during cell development [97, 98, 99, 100]. In contrast to the widespread use of chromobodies, the additional Nb‐based molecular machinery spreads poorly outside the laboratory that developed the technology. This might reflect the difficulty to extend the technology to other targets or to other cell types. Nevertheless, a few additional interesting and promising Nb‐based constructs emerged. For example, a bacterial circularly permutated dihydrofolate reductase (cpDHFR) enzyme was inserted close to the CDR3 of Nbs [101]. This did not prevent the Nb moiety from associating with its cognate Ag. However, the addition of nicotinamide adenine dinucleotide phosphate cofactor and trimethoprim inhibitor to cpDHFR induces conformational changes that prevent further interaction between the Nb and its target. This chemogenetic control of Ag recognition by Nbs apparently allows the investigation of the various biological processes. Furthermore, Nb fragments have been linked to light‐inducible heterodimerisation domains to form ‘optobodies’. Normally, these Nb fragments fail to assemble and recognise the Ag, but when exposed to blue‐light, the Nb assembles and binds to its Ag so as to suppress the endogenous function of the Ag [102]. Likewise, the Nb has been split and inserted into the solvent‐exposed β‐hairpin loop of larger proteins. The resulting ‘megabodies’ were mixed with the target of the Nb and used for cryo‐EM structure determination [103, 104]. All these genetic tricks demonstrate the flexibility exhibited by the Nb genes while producing functional novel proteins for innovative research investigations.

### Enzyme‐mediated conjugation

As mentioned earlier, the ‘Avi’ tag at the C‐terminal end of Nbs has been shown extremely practical for site‐specific biotinylating the Nb [74, 81]. Protein‐mediated ligation of Nbs can be accomplished by a variety of TagCatcher systems such as transglutaminases, tubulin tyrosine ligase or Sortase A. The Sortase A [105, 106] is a staphylococcal transpeptidase that naturally catalyses the anchoring of cell wall proteins to the peptidoglycan layer of the bacterial cell. This Sortase A enzyme recognises a pentapeptide tag (LPXTG) that is cleaved between T and G to form an acyl‐intermediate that is subsequently attacked by a nucleophilic probe. Of interest, proteins with N‐terminal glycines serve as such nucleophilic probes and can be obtained by first expressing a recombinant protein with a tobacco etch virus (TEV) cleavage tag followed by three glycines and the remainder of the target protein and then digesting this fusion with TEV protease [107]. Unluckily, protein ligations mediated by Sortase A reconstitute the original recognition sequence of the enzyme and serve therefore again as substrate for further reaction. This can be avoided by using a recombinant *Oldenlandia affinis* asparaginyl endopeptidase, OaAEP1, which can use incoming nucleophiles that generate cleavage‐resistant end products [108].

Interestingly, Nbs have been equipped with an LCTPSR peptide tag, also known as the aldehyde tag that is recognised by the formyl‐glycine generating enzyme (FGE) [109]. The FGE converts the sulphydryl group of the free cysteine into an aldehyde that can subsequently be reacted with various orthogonal nucleophiles forming site‐selectively Nb conjugates or with amino‐activated beads to form immune adsorbents [110, 111].

### Chemical crosslinking

Purified Nbs mixed with other proteins can be chemically crosslinked with formaldehyde, glutaraldehyde or other standard chemical bifunctional crosslinkers. Crosslinks will be mainly connected to the ε‐amino groups of lysines. Nbs have usually only 3–4 conserved lysines, luckily relatively distal from the CDR. It is a fast and simple chemistry that is, however, less appropriate if a lysine is present within the CDR. Furthermore, since it is a random crosslinking to an often bulky partner, the Ag‐binding capacity of the crosslinked Nb might be reduced [112].

Alternatively, an extra cysteine can be included at the C‐terminal end of the Nb, appropriately spaced by a short linker to maintain good production levels. After purification of the monomeric and dimeric Nb fractions, and a mild reduction step to make the sulphydryl available without reducing the intradomain disulphide bonds, maleimide decorated probes or drugs are efficiently conjugated [113].

## Applications where nanobodies make the difference

### Nanobodies as research tool

A versatile affinity reagent to be used as a research tool should be easy to generate and produce and it should be target‐specific. Sometimes it is helpful if the affinity reagent recognises all possible conformers of the target without interfering with its possible functions. However, it is equally important to have access to binders that recognise and fix one single conformer, thereby modulating the function of the target. Preferably, the affinity reagent should be functioning in different cellular compartments. Nbs like many other affinity reagents fit perfectly in this picture. Their high specificity for one particular conformation of the Ag makes that the crystallisation of the Nb‐Ag complex is highly successful, even for dynamic molecules [103]. As a result, well over 200 protein crystal structures were solved, of which the vast majority of proteins failed to have their structure determined at high resolution [114]. Nbs were also instrumental to identify the interaction side of subdomains of large proteins or to investigate in real time the protein–protein interactions within a cell and for superresolution microscopy [94, 115, 116]. The function of native enzymes has been knocked out or modified, even intracellularly, after direct Nb binding, by mediating the proteolysis of the Ag [97, 99], by its rerouting to other cellular compartments or by keeping the Ag at a location where it is not functioning [101, 117, 118]. Such functional knockouts are of significant interest in cell biology or in unveiling the life cycle of infectious pathogens. Also tracking and the study of the dynamics of the Ag inside cells became feasible with chromobodies or LlamaTags, obtained after intracellular expression of a Nb‐fluorescent protein gene construct [92, 119].

### Nanobodies in diagnostic applications

Many Nbs are at the core of both, *in vitro* and *in vivo* diagnostics. For the *in vitro* work, their specificity, low cost of goods, robustness and easy tailoring are well appreciated. As such, Nbs are being developed in affordable lateral flow assays to screen and test people and animals at remote places, or in field tests in absence of a cold chain to preserve fragile biochemicals [120]. Also, Nbs are introduced as capturing and detecting agents in immediate electrochemical tests to reach higher diagnostic sensitivity [121, 122, 123]. In addition, the robustness of Nbs to maintain their functionality under harsh chemical conditions was of key importance to enable the detection of food contaminants which were extracted in high concentrations of ‘protein‐denaturing’ methanol [124].

The requirements of the characteristics of the affinity reagents used for *in vivo* diagnostics are very different. To obtain a good contrast in noninvasive *in vivo* imaging, it is crucial to have a tool that is rapidly extravasating, diffusing deep into tissues without sticking to nontargeted cells, while the excess of material is rapidly eliminated from the body, preferably via the kidneys. This is exactly what Nbs seem to do, and therefore, Nbs appear to be an exquisite tool for SPECT/CT or PET/CT [125, 126, 127]. Besides the many preclinical tests, the Nb against HER2 breast carcinoma Ag passed successfully the phase I clinical trial [128].

### Nanobodies in therapy

Caplacizumab, a bivalent Nb construct against von Willebrand factor, is the first EMEA and FDA‐approved therapeutic that is life‐saving in acquired thrombotic thrombocytopenic purpura patients [129]. As the name suggests, this is a humanised Nb, although immunogenicity does not seem to occur even with wild‐type Nbs. Several additional Nb constructs directed against a broad range of autoimmune diseases reached various phases of clinical trials [130, 131].

Some of these Nbs are used as a building block into manifold constructs, to generate bispecific constructs such as BiTEs, where two Ags or cells are crosslinked *in situ* [84]. However, sometimes one of the composing Nbs is targeting albumin. While such multimeric Nb constructs exhibit an increased blood retention time, they have a reduced extravasation and a lower tissue penetration capacity [132].

By contrast, in the Nb‐based targeted radionucleotide therapy (TRNT) approach, the Nb is kept monomeric while conjugated either via a small chelator or directly to a radioactive therapeutic nuclide [133] (or coupled to a small drug into an antibody–drug conjugate) to preserve the fast extravasation and deep tissue diffusion. The drawback here is that such monomeric constructs are rapidly cleared from blood circulation through the kidneys, so that only a minor fraction of the injected material reaches the tumour.

Apart from the encouraging therapeutic results with Nb‐drug conjugates and Nb‐radionuclides, Nbs have also been introduced as chimeric Ag receptor constructs in single T cells (CAR‐T). Such Nb‐based CAR‐T constructs hold great promise in the field of adoptive cell‐based therapy [134, 135, 136].

Obviously, Nbs with specificity to toxins or viral pathogens have also been generated and tested against envenoming [137, 138] or for their antiviral capacity [139]. Interestingly, an Fc (hinge‐CH2, CH3) containing construct comprising multi‐Nb domains, each directed against hemagglutinins from influenza A and B viruses exhibited an enhanced cross‐reactivity and a broad protection in mice challenged with influenza [140].

### Nanobodies in biotech applications

Affinity chromatography relies on the specific adsorption of a particular target [110, 141]. It can be used to purify the target from a complex mixture, while sometimes it is only intended to remove an unwanted compound from a contaminated solution. In the former application, the affinity of the interaction should not be too strong as the target should be eluted under mild conditions. The high specificity and the adaptation of Nbs to obtain a directional immobilisation on an inert resin contribute to the success of Nb‐based affinity chromatography [110, 142].

The Nbs have also found their way towards the Agro‐biotech field. For example, Nbs directed against fungal glucosylceramide were shown to prevent the growth of *Botrytis cinerea* and to reduce the disease symptoms when this fungus was sprayed on tomato plants [143]. Likewise, a Nb against grapevine fanleaf virus coat protein confers strong resistance to this virus in transgenic *Nicotiana benthamiana* plants [144, 145]. A similar strategy was also followed with Nbs against broad bean mottle virus (BBMV) that upon transient expression in *Vicia faba* could immune protect the plant against BBMV infection [146].

Finally, an enterotoxigenic *E. coli*‐specific Nb was fused to the Fc regions of either IgA or IgG and expressed in plant seeds. A passive immunisation was obtained by feeding piglets with these seeds since the animals were apparently protected as witnessed by a significant gain in weight and a decline in shedding bacteria upon a bacterial challenge [147, 148].

## Perspectives

The naïve and certainly the synthetic Nb libraries are a good option to retrieve practical Nbs against virtual any target, especially when it is difficult to immunise or when Nbs are required against every possible epitope on the surface of the Ag. However, the immune Nb libraries against proteinaceous targets are at least as effective to retrieve very potent affinity reagents, even considering the potential epitope gaps that might occur during the time of immunisation. The proliferation of target‐specific B cells during the immunisation leading to an increased titre of affinity‐matured, Ag‐binding HCAbs and thus Nbs in the immune library facilitates the panning. This will allow an easier and more successful phenotypic screening, and it might even be developed in a strategy of target discovery. The Nb generation, especially from immune libraries, is and will remain for a long time a highly valued tool to determine the structure and the mechanism of action of many proteins.

The Nbs will find their way in several diagnostic tests, especially when the fragility of classical antibodies or other affinity reagents prevent their use under harsh conditions (extreme temperature or chemical denaturants). The small size allowing a higher density of the target detecting entities, in conjunction with the engineering of the Nbs to obtain a directional orientation to reach a higher functional concentration, will form the basis of increased diagnostic sensitivity, further supported with the low a‐specific adsorption of unwanted molecules. The low cost of goods and the robustness of Nbs will be a key feature for the development of cheap and sensitive diagnostic kits, either as LFA or as electrochemical detection assays. Alternatively, the ideal biodistribution properties of Nbs render these molecules perfect tracers for diseased tissues by noninvasive *in vivo* imaging.

Finally, Nbs will be at the core of many future therapeutics. These Nbs can be used as autonomous entities where the intravenously administered Nbs, or Nbs conjugated to small drugs or nuclides, will rapidly extravasate and penetrate deep into tissues, while excess toxic material will be eliminated via the kidneys. However, the fast elimination from the body makes that the Nb load on the diseased site is only a minor fraction of what is injected. In this respect, it might be a better strategy to fuse the therapeutic Nb to a Nb against serum albumin or any other abundant serum protein to increase the retention time in the patient. However, this will be at the cost of fast extravasation and deep tissue penetration. The choice for monomeric TRNT Nbs or multimeric CAR‐T or BiTE Nb constructs will be dictated to a large extent by the disease.

## Conflict of interest

SM is consultant for KisoJi Biotechnology Inc., Shenzhen Pregene Biopharma Co, Ltd. and Kang Yuan Biomedical Technologies.
